# Narrow Linewidth All-Optical Microwave Oscillator Based on Torsional Radial Acoustic Modes of Single-Mode Fiber

**DOI:** 10.3390/mi16010097

**Published:** 2025-01-15

**Authors:** Wen Wang, Wenjun He, Xinyue Fang, Yi Liu, Yajun You, Mingxing Li, Lei Yu, Qing Yan, Yafei Hou, Jian He, Xiujian Chou

**Affiliations:** 1 Key Laboratory of Instrumentation Science and Dynamic Measurement Ministry of Education, North University of China, Taiyuan 030051, China; wangwen202308@163.com (W.W.); 17860737007@163.com (X.F.); 19834538941@163.com (M.L.); yulei13835520751@163.com (L.Y.); 15525490669@163.com (Q.Y.); houyafei_tl@163.com (Y.H.); drhejian@nuc.edu.cn (J.H.); 2 Shanxi Key Laboratory of Ferroelectric Physical Micronano Devices and Systems, North University of China, Taiyuan 030051, China; yajunyou@nuc.edu.cn; 3 Thinvent Digital Technology Co., Ltd., 681 Torch Avenue, High-Tech Industrial Development Zone, Nanchang 330096, China; 4 School of Aerospace Engineering, North University of China, Taiyuan 030051, China

**Keywords:** torsional radial acoustic mode, single longitudinal mode, all-optical microwave oscillator, narrow linewidth, forward stimulated Brillouin scattering

## Abstract

A Hz level narrow linewidth all-optical microwave oscillator based on the torsional radial acoustic modes (TR_2,m_) of a single-mode fiber (SMF) is proposed and validated. The all-optical microwave oscillator consists of a 20 km SMF main ring cavity and a 5 km SMF sub ring cavity. The main ring cavity provides forward stimulated Brillouin scattering gain and utilizes a nonlinear polarization rotation effect to achieve TR_2,7_ mode locking. By combining the sub ring cavity with the main ring cavity and utilizing the Vernier effect, the TR_2,7_ mode microwave photonic single longitudinal mode (SLM) output can be ensured. Meanwhile, the 6.281 Hz narrow linewidth of the TR_2,7_ mode is achieved by reducing the intrinsic linewidth of the passive resonant cavity. The acoustic mode suppression ratio and side mode suppression ratio of the TR_2,7_ mode were 43 dB and 54 dB, respectively. The power and frequency fluctuations of within 40 min were approximately ±0.49 dB and ±0.187 kHz, indicating good stability. At a frequency offset of 10 kHz, the TR_2,7_ mode had a low phase noise value of −110 dBc/Hz. This solution can be used in various fields, such as high-precision radar detection, long-distance optical communication, and high-performance fiber optic sensing.

## 1. Introduction

Narrow linewidth and low noise single longitudinal mode (SLM) all-optical microwave oscillators have broad application prospects in the wireless communication, fiber optic sensing, and radar detection fields [1,2,3]. Forward stimulated Brillouin scattering (FSBS) in optical fibers can be well applied in all-optical microwave oscillators due to its low threshold and narrow linewidth characteristics. Forward spontaneous Brillouin scattering in optical fibers, also known as guided acoustic wave Brillouin scattering (GAWBS), is caused by transverse acoustic modes, where the signal light and pump light propagate in the same direction along the optical fiber. The transverse acoustic modes involved in the GAWBS process are divided into radial acoustic modes (R_0,m_) and torsional radial acoustic modes (TR_2,m_) based on displacement equations. The R_0,m_ mode expands or compresses simultaneously in all directions and is a polarization insensitive radial acoustic mode, which only modulates the phase of light [4]. The TR_2,m_ mode is polarization sensitive and forms two degenerate modes in different combinations, namely the TR_2,m_ mode (90°/0° vibration form) and TR_2,m_ mode (45°/−45° vibration form). The R_0,m_ mode and TR_2,m_ mode (90°/0° vibration form) can only modulate the phase of light and are classified as pure phase modulation. The TR_2,m_ mode (45°/−45° vibration form) can modulate the polarization state and phase of incident light [5]. There are many studies on the R_0,m_ mode and TR_2,m_ mode in GAWBS [6,7], but there are relatively few applications.

The TR_2,m_ mode has important research value in the field of microwave photonic oscillation due to its advantages in polarization sensitivity and relatively more complex tensor features. The ways to generate FSBS microwave photonic oscillation include optoelectronic oscillators (OEO) and passive mode-locked fiber oscillators [8,9,10,11,12,13,14,15,16,17]. Among them, OEO requires complex microwave photonic oscillation generation devices such as optoelectronic modulators and optoelectronic converters. In contrast, passive mode locking technology has received widespread attention and research both domestically and internationally due to its advantages of a simple structure, low cost, and high pulse power. Compared with other passive mode locking methods, nonlinear polarization rotation (NPR) technology is widely studied for its ease in obtaining high peak power pulses and lower production costs. K. Tamura et al. [8] first used NPR technology to obtain sub picosecond mode-locked pulses with a repetition rate of 42 MHz. M.S. Kang et al. [9] used NPR technology to generate high repetition rate (1.80 GHz) passively mode-locked EDF ring lasers. Jia et al. [10] used NPR to generate passive harmonic mode-locked pulses with a basic repetition rate of 3.178 MHz in thulium doped fiber lasers. Shang et al. [11] designed an all fiber erbium-doped mode-locked laser with a fundamental frequency of 21.65 MHz using NPR technology. S.N. Ma et al. [12] combined FSBS and NPR techniques to achieve R_0,m_ mode oscillation output. Therefore, NPR technology is a valid way to generate microwave photonic oscillation based on FSBS. However, most research on FSBS microwave photonic oscillation has focused on R_0,m_ modes, and studies on polarization sensitive TR_2,m_ modes only exist in GAWBS, with almost no research on TR_2,m_ mode microwave photonic generation in FSBS. Consequently, we can utilize the polarization sensitivity advantage of TR_2,m_ mode combined with NPR technology to achieve FSBS microwave photonic oscillation in TR_2,m_ mode. In addition, SLM Brillouin fiber lasers or oscillators are widely used in various fields such as microwave photonic generation [18] due to their inherent stability and narrow linewidth output characteristics [19,20]. Based on the Vernier effect, using double or multi ring structures to increase the free spectral range (FSR) is a very effective method for achieving SLM output [21,22,23,24,25]. There have been some previous studies using FSBS to achieve SLM microwave photonic generation [26,27]. Similarly, most studies on FSBS SLM have focused on R_0,m_ modes, with little research on polarization sensitive TR_2,m_ modes. This is because TR_2,m_ mode scattering is relatively weak, and high side mode suppression ratio (SMSR) TR_2,m_ mode SLM oscillation research is difficult to achieve. Fortunately, these previous studies have provided the possibility for achieving high coherence, high SMSR, and high stability of TR_2,m_ mode SLM output in this research.

In this article, NPR mode locking technology was used to achieve TR_2,7_ mode locking. On the basis of realizing multi-mode mode locking in a single ring cavity, we continued to utilize the dual ring Vernier effect to achieve TR_2,7_ mode single mode locking and SLM oscillation output. Compared to OEO that can generate microwave signals [15,16,17], the proposed scheme exhibits higher SMSR and the effect of SLM is better. Furthermore, in contrast to prior research on all-optical microwave oscillators based on radial acoustic modes [22,27], this scheme demonstrates a superior acoustic mode suppression ratio, SMSR, and stability performance. In this scheme, the all-optical microwave oscillator consists of a 20 km single-mode fiber (SMF) main ring and a 5 km SMF sub ring. The main ring provides FSBS gain and implementation mode locking, while the combination of the sub ring cavity and the main ring cavity validates TR_2,7_ mode SLM output based on the Vernier effect. By reducing the inherent linewidth of the passive resonant cavity, the 6.281 Hz narrow linewidth TR_2,7_ mode SLM output could be achieved. The pump threshold power was 16.5 dBm. The acoustic mode suppression ratio and SMSR could reach 43 dB and 54 dB, respectively. Within the 40 min range, the power and frequency fluctuations were approximately ±0.49 dB and ±0.187 kHz, respectively. At a frequency offset of 10 kHz, the TR_2,7_ mode has a low phase noise value of −110 dBc/Hz. Although the positive dispersion caused by a long SMF will lead to pulse broadening, the saturable absorption effect of the NPR device in this scheme will narrow the pulse to achieve a certain degree of balance, ultimately delivering excellent performance [28,29,30]. This scheme is of great significance for research on forward Brillouin scattering applications, especially fiber sensing applications. In the field of fiber optic sensing, the TR_2,m_ mode can enhance the function of forward Brillouin scattering fiber sensors with its more complex tensor characteristics, achieving smaller sensing experimental errors [31].

## 2. Experimental Setup and Basic Principles

**Figure 1:**
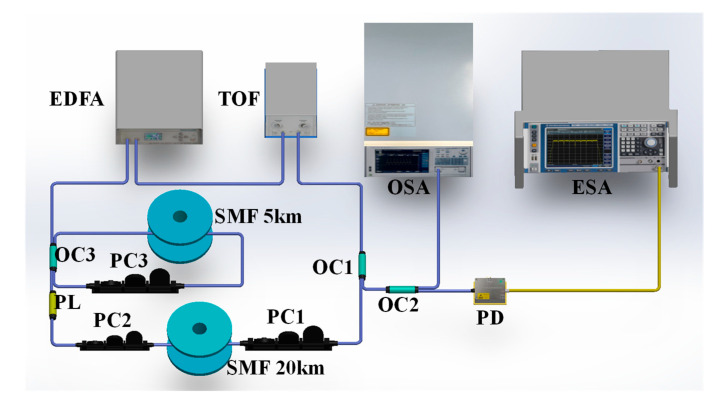
Experimental setup of narrow linewidth all-optical microwave oscillator based on torsional radial acoustic modes. EDFA: Erbium-doped fiber amplifier; TOF: Tunable filter; OC: Optical coupler; PC: Polarization controller; SMF: Single-mode optical fiber; PL: Polarizer; PD: Photodetector; OSA: Optical spectral analyzer; ESA: Electronic spectrum analyzer.

The passive mode-locked narrow linewidth SLM all-optical microwave oscillator device based on torsional radial acoustic modes is shown in Figure 1. It consists of a 20 km SMF main ring and a 5 km SMF sub ring. In the main ring cavity, a self-excited erbium-doped fiber amplifier (EDFA, EDFA-C-25-FA-B) is used to provide a maximum pump power of 25 dBm. A tunable optical filter (TOF, XTM-50-SCL-S-M) is used to select the appropriate GAWBS pump wavelength. A polarizer (PL) and two polarization controllers (PC) form an NPR component to achieve passive mode locking. A 20 km long SMF is used as an FSBS gain fiber for selective amplification of longitudinal modes. After passing through the PL in the annular cavity clockwise, linearly polarized light becomes elliptically polarized light through PC1. Due to nonlinear effects, polarization state rotation occurs. By fine-tuning PC2, when the light signal passes through the PL again, the peak transmittance of the pulse is high, forming a similar saturable absorber. In a stable state, a cavity round-trip frequency pulse f is generated that is related to the total length L of the cavity. When the harmonic frequency of the pulse train approaches the FSBS sound frequency of the fiber, the refractive index modulation is enhanced and sequentially acts on the driving pulse, achieving an FSBS enhanced passive mode-locked oscillator. The FSBS generated by the main ring cavity circulates back and forth in the sub ring cavity through a 50/50 optocoupler (OC3). A sub ring cavity with 5 km SMF and PC3 is adopted to achieve effective FSR alignment of the double cavity, thus achieving SLM operation of FSBS Stokes. Both 20 km SMF and 5 km SMF are placed in a 25 °C thermostat with a resolution of 0.2 °C, ensuring a constant temperature environment. The 10% of ports in 90:10 OC1 is used as output. Afterwards, it can be divided into two parts by 90:10 OC2, and 90% of the FSBS Stokes light is monitored by an optical spectral analyzer (OSA, AQ6370D-02EN) with a resolution bandwidth (RBW) of 0.02 nm. The other 10% of the FSBS Stokes light is converted into an electrical signal by a photodetector (PD) and monitored by a 0.05 kHz RBW electrical spectrum analyzer (ESA, FSV3030).

**Figure 2:**
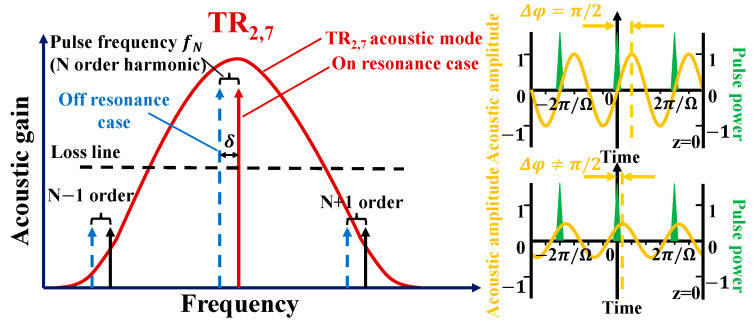
TR_2,7_ mode microwave photonic generation based on nonlinear polarization rotation effect.

In Figure 2, the FSBS enhance passive mode locking oscillator should satisfy the requirement that the difference δ between the repetition rate $f_{N}$ (an integer multiple of f) and the acoustic resonance frequency TR_2,m_ (m = 1, 2, 3...) is almost equal to zero, where $f_{N}=Nc/nL$ (N = 1, 2, 3), n is the effective refractive index of SMF, c is the speed of light in vacuum, and L is the length of the cavity. In our experiment, it is known that the TR_2,7_ of SMF is about 139 MHz, and the oscillator cavity length is designed to be L = 20 km. Therefore, when N = 13,605 and f = 0.0102 MHz, $f_{N}$ is close to TR_2,7_ mode frequency (139 MHz), satisfying the matching conditions. When the pulse energy $E_{p}$ reaches the threshold, the optical pulse modulates the refractive index through the electrostriction effect, which amplifies the amplitude of the sound wave. The FSBS process enhances the light pulse with a frequency of $f_{N}$ and suppresses other frequencies. The sound waves can be represented as [26]:

(1) $$\Delta \varepsilon_{r}(z,t,r,\theta )=\frac{\gamma_{e}^{2}\left|Q\right|E_{p}\rho_{a}(r,\theta )e^{i(\Omega_{2m}t-qz-\Delta \varphi )}}{4\pi ncA\rho_{0}\sqrt{4\delta^{2}+{\left(\Gamma_{B}\right)}^{2}}}.$$

where $\gamma_{e}$ is the electrostriction coefficient, Q is the overlapping integral between the acoustic mode and the electrostriction stress field, $\rho_{a}(r,\theta )$ is the normalized mode profile, q is the propagation constant, A is the effective mode area, $\rho_{0}$ is the density of silicon dioxide, and $\Gamma_{B}$ is the Brillouin linewidth of the acoustic mode. Δφ is the relative phase shift between the sound wave and its driving pulse train, and is given by the following equation:

(2) $$\Delta \varphi =\mathrm{arccot}(-\frac{2\delta }{\Gamma_{B}}),0\leq \Delta \varphi \leq \pi .$$

According to the phase matching conditions, when Δφ is within the range of (0, π), sound gain occurs, and when Δφ=π/2 (Δ = 0), the sound wave amplitude reaches its maximum value. In this study, the TR_2,7_ mode was selected due to its highest theoretical scattering efficiency [5]. This selection enhances the passive mode locking output with a higher SMSR. TR_2,7_ mode microwave photonic oscillation can be achieved by enhancing FSBS passive mode locking and adjusting the PCs to match the phase matching conditions.

**Figure 3:**
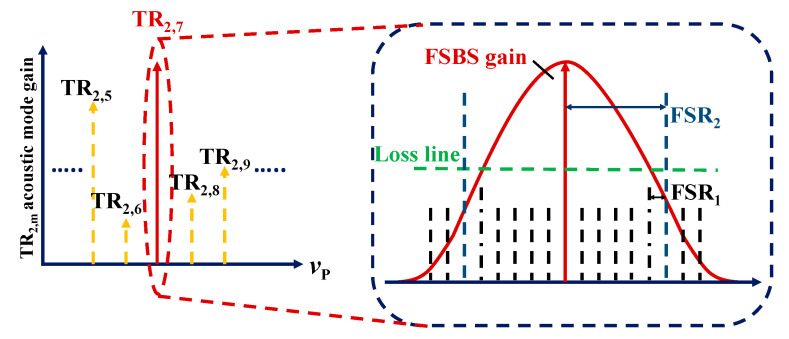
Principle of TR_2,7_ mode microwave photonic SLM generation.

Utilizing the Vernier effect, the effective FSR of a double ring cavity can be obtained by the minimum common multiple of the main ring cavity and the sub ring cavity:

(3) $$FSR=n_{1}FSR_{1}=n_{2}FSR_{2}.$$

where FSR_1_ and FSR_2_ correspond to the 20 km SMF main ring cavity and the 5 km SMF sub ring cavity, respectively:

(4) $$FSR_{m}=c/nL_{m},(m=1,2).$$

where L_m_ (m = 1,2) is the ring length of the main and sub ring cavities, and n = 1.4682 is the effective refractive index of SMF. The FSR_1_ and FSR_2_ are about 10 kHz and 41 kHz respectively. As shown in Figure 3, when the pump light from $v_{p}$ is injected into SMF, it excites FSBS. If the FSR of the double ring cavity exceeds the gain bandwidth of FSBS and the gain is greater than the loss, FSBS can only oscillate at frequencies that satisfy both resonance conditions of the main ring cavity and sub-ring cavity. The yellow dashed arrow in Figure 3 represents the upper sideband of each FSBS, while the red solid arrow represents the TR_2,7_ mode FSBS SLM achieved by adjusting the PCs in the double ring cavity.

The TR_2,7_ mode microwave photonic linewidth narrowing of the all-optical microwave oscillator is achieved by reducing the inherent linewidth of the passive resonant cavity, which has a great linewidth reduction effect. The formula for calculating the linewidth of the all-optical microwave oscillator has been derived [22,32,33] as follows:

(5) $$\Delta v_{s}=\frac{\Delta v_{c}}{{\left(1+\frac{\pi \Delta v_{B}}{-cInR/nL}\right)}^{2}}=\frac{c(1-K_{r})}{nL\pi \sqrt{K_{r}}{\left(1+\frac{\pi \Delta v_{B}}{-cInR/nL}\right)}^{2}}.$$

where $\Delta v_{c}=c(1-K_{r})/nL\pi \sqrt{K_{r}}$ is the intrinsic linewidth of a passive resonant cavity, $K_{r}\approx 0.98$ is the strength coupling coefficient, c is the speed of light in a vacuum, L = 20 km is the length of the fiber ring cavity, and n = 1.4682 is the refractive index of SMF. The effective FSR of the single ring cavity is 10 kHz, $\Delta v_{B}\approx 2MHz$ is the TR_2,7_ mode gain bandwidth, and R=0.5×0.9=0.45 is the optical amplitude feedback coefficient of the ring cavity. By substituting the above parameter values into Equation (5), we can roughly estimate that the TR_2,7_ mode microwave photonic theoretical linewidth is $10^{-4}$ Hz.

## 3. Results

### 3.1. Heterodyne Spectra of All-Optical Microwave Oscillator

**Figure 4:**
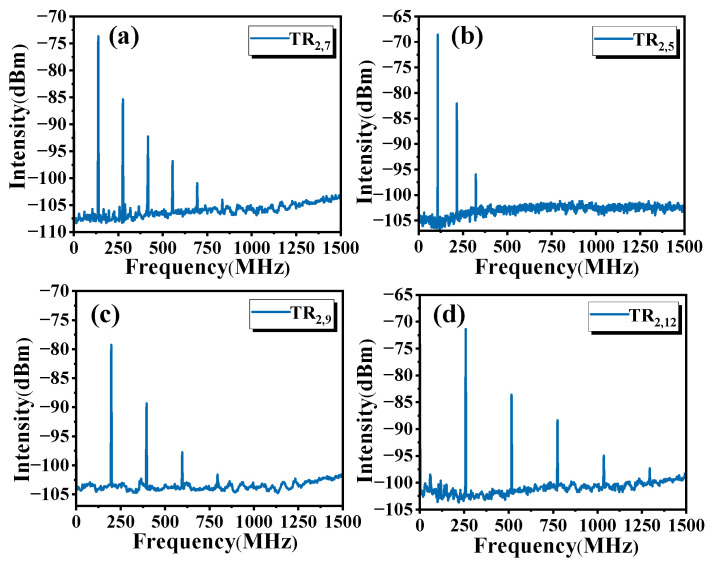
Mode locking heterodyne spectra of different TR_2,m_ modes in a 20 km single ring cavity with 1.5 GHz span. (**a**) TR_2,7_ mode; (**b**) TR_2,5_ mode; (**c**) TR_2,9_ mode; (**d**) TR_2,12_ mode.

NPR passive mode-locking in TR_2,m_ acoustic modes can be achieved by carefully adjusting the PCs in the single ring cavity. As shown in Figure 4, we have implemented mode locking for TR_2,m_ modes with high theoretical scattering efficiency, such as TR_2,7_ (139.0 MHz), TR_2,5_ (107.4 MHz), TR_2,9_ (199.3 MHz) and TR_2,12_ (258.8 MHz). These acoustic modes can produce high experimental intensity mode locking. Among them, the TR_2,7_ mode has the highest theoretical scattering efficiency value [5]. In addition to high experimental intensity, the TR_2,7_ mode is easier to realize mode locking and generate more higher-order modes than the other modes in our experiment, which has important research value. Therefore, in the subsequent experiment, we chose the TR_2,7_ mode as an example to carry out the research.

**Figure 5:**
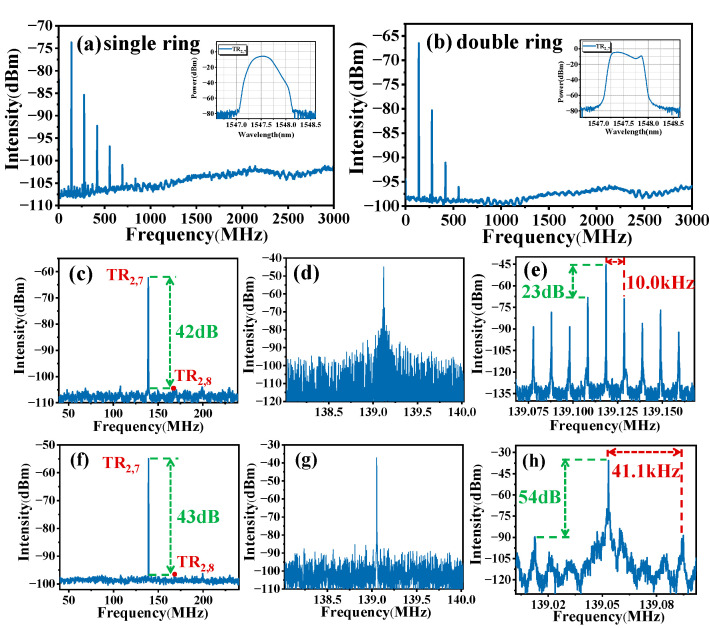
Heterodyne spectra of TR_2,7_ mode microwave photonic oscillation in different spans. (**a**,**c**–**e**) In the 20 km single ring cavity with 3 GHz, 200 MHz, 2 MHz, and 100 kHz spans; (**b**,**f**–**h**) in the double ring cavity composed of the 20 km main ring cavity and 5 km sub ring cavity with 3 GHz, 200 MHz, 2 MHz, and 100 kHz spans.

In the double ring cavity, SLM output can be achieved under the Vernier effect. Figure 5a,b illustrate the heterodyne spectra within 3 GHz span in single ring and double ring cavities, respectively, with a RBW of 1 kHz. The upper right graphs show the spectral detection with a central wavelength of 1547.6 nm. The peak at 139 MHz and its integer multiples correspond to the mode locking at the 13,605 (N) harmonic of the fundamental cavity frequency (0.0102 MHz). Figure 5c–e and Figure 5f–h show the heterodyne spectra at 200 MHz, 2 MHz and 100 kHz spans in single ring and double ring cavities, respectively. Among them, the RBW for Figure 5e,h is set at 50 Hz. The acoustic mode suppression ratios presented in Figure 5c,f are measured at 42 dB and 43 dB, respectively, surpassing the values reported in prior studies [22]. Comparing Figure 5d with Figure 5g, it can be seen that the TR_2,7_ mode microwave photonic adjacent longitudinal modes are well suppressed when there is a sub ring cavity. When the span is reduced to 100 kHz and there is no sub ring cavity, the spacing between TR_2,7_ mode microwave photonic adjacent longitudinal modes is 10.0 kHz in Figure 5e, almost corresponding to the FSR of 20 km SMF, and the SMSR is 23 dB. Moreover, the SMSR of the double ring cavity is 54 dB, as shown in Figure 5h, which is 31 dB higher than that of the single ring cavity, achieving a good SLM effect. The TR_2,7_ mode microwave photonic adjacent longitudinal mode spacing is 41.1 kHz, almost corresponding to the FSR of the double ring cavity.

### 3.2. Linewidth Measurement of All-Optical Microwave Oscillator

**Figure 6:**
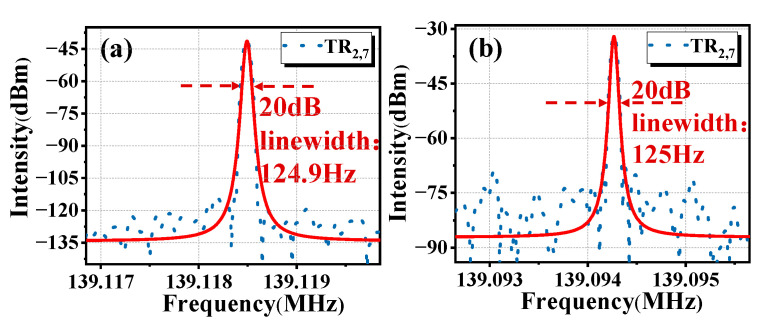
Linewidth measurement results. (**a**) In the single ring cavity; (**b**) in the double ring cavity.

In Figure 6, we have measured the linewidth at −20 dB of a single ring cavity and double ring cavity when the span is 3 kHz and the RBW is 50 Hz, and the results are 124.9 Hz and 125 Hz, respectively. The true linewidth value is $2\sqrt{99}$ times the −20 dB linewidth value, so the true linewidth value is 6.276 Hz and 6.281 Hz, respectively. The linewidth value remains relatively unchanged. However, according to Equation (5), the theoretical linewidth of the all-optical microwave oscillator can be computed as $10^{-4}$ Hz, which greatly exceeds the 1 Hz resolution accuracy of the ESA. The measured linewidth being greater than the theoretical value may be due to system noise and unstable operation [34].

### 3.3. Threshold Measurement of All-Optical Microwave Oscillator

**Figure 7:**
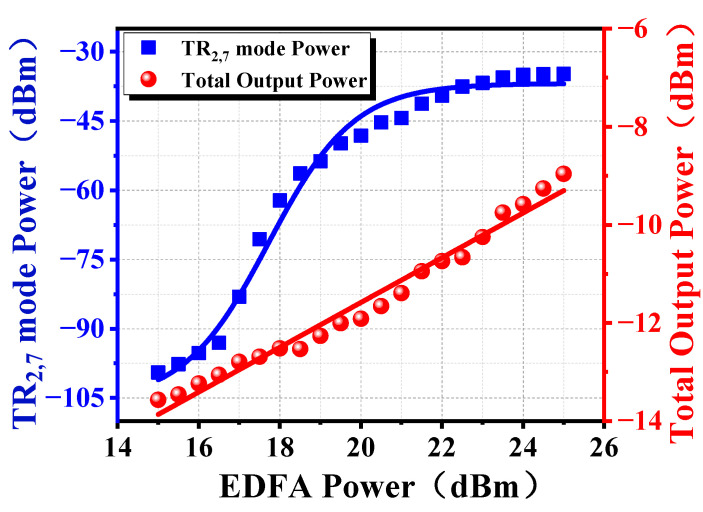
Threshold measurement results of double ring all-optical microwave oscillator.

Figure 7 shows the threshold measurement of a double ring all-optical microwave oscillator. In the experiment, the total output power of the all-optical microwave oscillator is measured by OSA, and the TR_2,7_ mode microwave photonic output power is measured by ESA. Obviously, the total output power of the all-optical microwave oscillator increases linearly with the EDFA pump light power. Furthermore, the TR_2,7_ mode microwave photonic output power gradually increases before the pump power reaches 16.5 dBm, then sharply increases when it reaches 16.5 dBm, and subsequently stabilizes. The experiment indicates that the threshold of the double cavity all-optical microwave oscillator is about 16.5 dBm pump power.

### 3.4. Stability Measurement of All-Optical Microwave Oscillator

**Figure 8:**
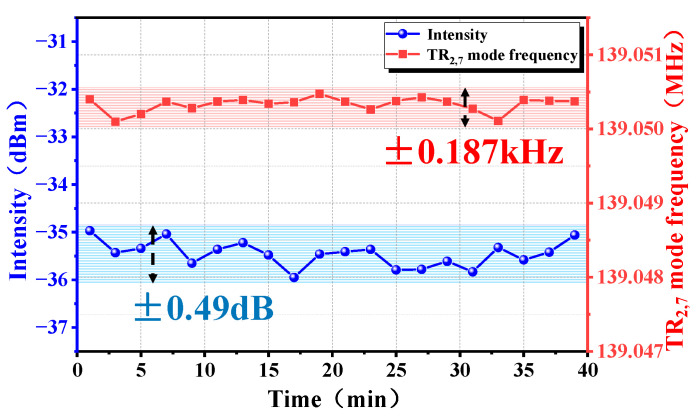
Stability measurement results of TR_2,7_ mode microwave photonic oscillation.

Through long-term testing (maintained for about 40 min here), we have demonstrated that our designed system is stable. To ensure the accuracy of the measurement results, the SMF in the dual ring cavity was placed in a thermostat at 25 °C with a resolution of 0.2 °C. As shown in Figure 8, the TR_2,7_ mode microwave photonic power fluctuation is ±0.49 dB, and this may be caused by the TR_2,7_ mode microwave photonic sensitivity to environmental temperature and noise. In terms of frequency stability, the TR_2,7_ mode microwave photonic frequency variation fluctuates within a range of ±0.187 kHz, far less than twice the FSR, and there is no mode hopping phenomenon.

### 3.5. SSB Phase Noise Measurement of All-Optical Microwave Oscillator

**Figure 9:**
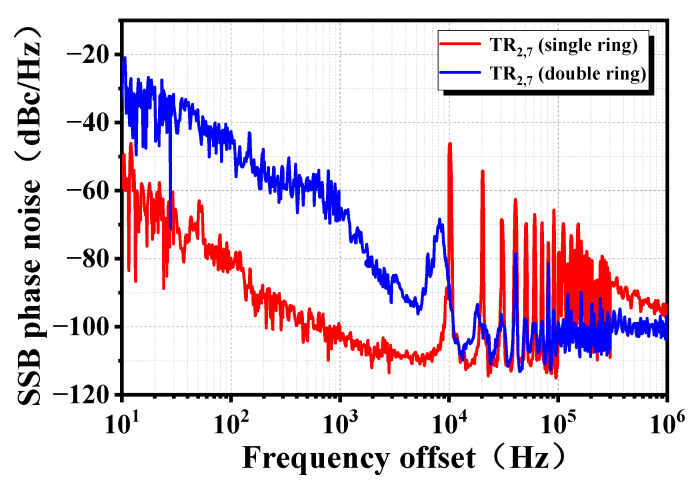
SSB phase noise measurement results of TR_2,7_ mode microwave photonic signals in single ring and double ring cavities, respectively.

The TR_2,7_ mode microwave photonic single sideband (SSB) phase noise in a single ring and double ring cavity is measured using ESA and its optional components, as shown in Figure 9. When the frequency offset is around 10 kHz, the double ring TR_2,7_ mode has an optimal phase noise value of −110 dBc/Hz. From the two curves in the Figure 9, multiple peaks can be observed starting from 10 kHz and 41 kHz, corresponding to the FSR of the single ring cavity and the double ring cavity, respectively. By comparing these relative peak values, it can be seen that the edge modes of the double ring cavity are well suppressed compared to those of the single ring cavity. In addition, due to the relatively large influence of the environment on the dual ring structure, the peak of the dual ring at approximately 8 kHz is caused by vibration interference in the laboratory [35,36]. Therefore, the dual ring cavity all-optical microwave oscillator can generate excellent microwave photonic signals.

## 4. Conclusions

In summary, a narrow linewidth all-optical microwave oscillator based on the torsional radial acoustic modes of a single-mode fiber is proposed. The all-optical microwave oscillator consists of a 20 km SMF main ring cavity and a 5 km SMF sub ring cavity. The main ring utilizes the NPR effect to achieve TR_2,7_ mode locking and provide FSBS gain. Meanwhile, the combination of the sub ring and the main ring ensures TR_2,7_ mode SLM output based on the Vernier effect. A 6.281 Hz narrow linewidth TR_2,7_ mode SLM microwave photonic oscillation is achieved. The acoustic mode suppression ratio and SMSR are 43 dB and 54 dB, respectively, and the EDFA pump threshold power is 16.5 dBm. The TR_2,7_ mode microwave photonic power and frequency fluctuations within 40 min are approximately ±0.49 dB and ±0.187 kHz, indicating high stability. The TR_2,7_ mode has an optimal phase noise value of −110 dBc/Hz when the frequency offset is about 10 kHz. This research can be widely applied in fields such as fiber optic sensing, wireless communication, radar detection, spectral analysis, etc., and has high research value.

## Data Availability

The data that support the findings of this study are available upon reasonable request from the authors.
